# Association of Respiratory Tuberculosis with Incident Bone Fracture: Bridging the Tuberculosis Airway Infection and the Osteoporotic Bone

**DOI:** 10.1371/journal.pone.0168673

**Published:** 2016-12-22

**Authors:** Jun-Jun Yeh, Yu-Chiao Wang, Che-Chen Lin, Cheng-Li Lin, Wu-Huei Hsu

**Affiliations:** 1 Ditmanson Medical Foundation Chia-Yi Christian Hospital, Chiayi, Taiwan; 2 Chia Nan University of Pharmacy and Science, Tainan, Taiwan; 3 Meiho University, Pingtung, Taiwan; 4 Management Office for Health Data, China Medical University Hospital, Taichung, Taiwan; 5 College of Medicine, China Medical University, Taichung, Taiwan; 6 Graduate Institute of Clinical Medical Science and School of Medicine, College of Medicine, China Medical University, Taichung, Taiwan; 7 Division of Pulmonary and Critical Care Medicine, Department of Internal Medicine, China Medical University Hospital, Taichung, Taiwan; Garvan Institute of Medical Research, AUSTRALIA

## Abstract

**Objective:**

The relationship between respiratory tuberculosis (RT) and incident fragility fracture and osteoporosis/fragility fracture in the general population is not well determined; therefore, we conducted a nationwide cohort study to investigate this relationship.

**Methods:**

We used the National Health Insurance Research Database of Taiwan to identify 6612 newly diagnosed patients with RT (RT cohort) and 13220 patients without RT (non-RT cohort) from 1999 to 2005. The mean durations of follow-up were (6.73 ± 4.00 years, 8.11 ± 3.24 years) in the (RT cohort, non- RT cohort); respectively. The occurrence of incident fragility fracture and osteoporosis/fragility fracture were followed up until the end of 2011. The adjusted hazard ratios (aHRs) and 95% confidence intervals (CIs) and 98% CIs of incident fragility fracture and osteoporosis/fragility fracture were estimated using the multivariable Cox proportional hazard model after adjusting for age, sex, occupation, drug use, and comorbidities.

**Results:**

A Cox proportional hazards regression analysis was performed and showed the aHRs of [incident fragility fracture; osteoporosis/fragility fracture] were [1.69 (95% CI = 1.26–2.28, 98% CI = 1.18–2.44); 1.42 (95% CI = 1.25–1.61, 98% CI = 1.21–1.65)] between the RT and non-RT cohorts. Regarding the sex, the aHRs of the [incident fragility fracture; osteoporosis / fragility fracture] were [1.57 (98% CI = 1.10–2.23, 98% CI = 1.02–2.41); 1.15 (95% CI = 0.97–1.36, 98% CI = 0.94–1.41)] in the men. The aHRs of the RT cohort without oral steroid use in the [incident fragility fracture; osteoporosis / fragility fracture] were [1.87 (95% CI = 1.20–2.90, 98% CI = 1.09–3.19); 1.41 (95% CI = 1.19–1.67, 98% CI = 1.14–1.74)].

**Conclusion:**

The RT associated with the incident fragility fracture, either in men or absence of oral steroid use.

## Introduction

Osteoporosis is the most common type of bone disease [1] and is a major public health problem [2]. Osteoporosis increases the risk of breaking a bone [3]. Approximately 50% of women older than 50 years were predicted to fracture their hip, wrist, or vertebra (bone of the spine) during their lifetime. Studies have reported that one out of 4 osteoporotic hip fractures resulted in long-term nursing home care, and half of these patients are unable to walk without assistance and have a 24% increased risk of death within one year [4,5].

Parathyroid hormone (PTH) stimulates osteoblasts, which increase the production of receptor activator of nuclear factor kappa-B ligand (RANKL) [6]. Hematopoietic cell precursors stimulated by macrophage colony-stimulating factor (M-CSF) facilitate osteoclast production that expresses RANK [6]. Osteoblasts also produce a decoy receptor called osteoprotegerin (OPG) that binds to RANKL and prevents RANKL and RANK interaction [7]. Estradiol increases the production of OPG to diminish bone resorption. Glucocorticoids stimulate RANKL expression and inhibit OPG synthesis by osteoblasts to enhance osteoclast proliferation and differentiation, leading to bone resorption [8].

Patient profiles at a bone health and osteoporosis prevention service center in Ireland revealed that only 13% of patients did not present with comorbidities [9]. Osteoporosis has been reported to be associated with hyperlipidemia, hypertension, diabetes, ischemic heart disease, end-stage renal disease [10], and liver cirrhosis [11]. Infections such as hepatitis C virus (HCV) infection are also risk factors of osteoporosis [11]. Osteoporotic changes [12] in tuberculosis arthritis were observed in previous report [13]. However, the respiratory tuberculosis (RT) intervention associated with bone fracture among patients with or without comorbidities is not found in literature.

## Methods and Materials

### Study population

**Fig 1:**
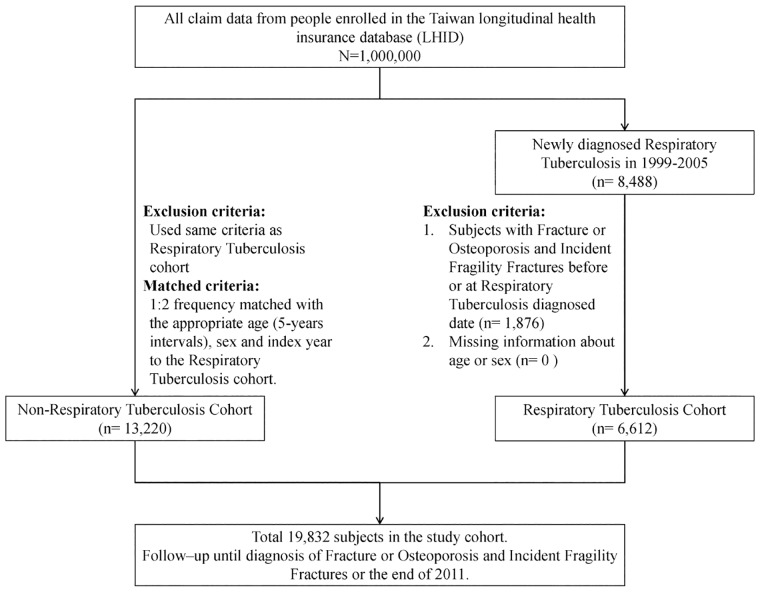
Flow chart of the selection of study participants.

The patient selection process used in our study is illustrated in Fig 1. We conducted a retrospective population-based cohort study to investigate the association between RT and risk of incident bone fracture and osteoporosis / fragility fracture. The diagnosis and treatment of the RT is under the strict control of the Centers for Disease Control in Taiwan for avoiding pulmonary tuberculosis (PTB) transmission [14]. The coding of the RT is after the consensus of the chest physician, infection specialist and the well-trained coder. The RT cohort follow up by the public nurse and chest physician or infection specialist. During the study period (1999–2005), we identified newly diagnosed patients with RT (ICD-9-CM 011–012) as the RT cohort, and the initial date of RT diagnosis served as their index date. We randomly selected comparison participants (individuals without RT) from the LHID as the non-RT cohort and frequency-matched the participants by age and sex at a ratio of 1:2. The index date of the non-RT cohort was randomly assigned a month and day with the same year as the matched cases. We excluded individuals with missing data such as date of birth, sex, and known history of osteoporosis / fragility fracture before the baseline from the study.

### Outcome and comorbidities measurement

The primary endpoint of our study was the occurrence of fragility fracture (ICD-9-CM 733.1). The secondary endpoint was osteoporosis /fragility fracture (pathology fractures; ICD-9-CM 733.0, 733.1) [15–17]. All participants were followed up from the index date to the date when the participants stopped claiming insurance and showing signs of incident fragility fracture, osteoporosis / fragility fracture or until December 31, 2011.

Comorbidities were defined as the patient having used medical services for hyperlipidemia (ICD-9-CM 272), hypertension (ICD-9-CM 401–405), diabetes (ICD-9-CM 250), pneumonia (ICD-9-CM 480–488), liver cirrhosis (ICD-9-CM 571), ischemia heart disease (IHD, ICD-9-CM 410–414), end-stage renal disease (ESRD, ICD-9-CM 585), hyperparathyroidism (ICD-9-CM 252.0), celiac disease (ICD-9-CM 579.0), Crohn’s disease (ICD-9-CM 555.0, 555.1, 555.2, and 555.9), alcohol-related illness (ICD-9-CM 291, 303, 305, 571.0, 571.1, 571.2, 571.3, and 790.3), stroke (ICD-9-CM 430–438), chronic obstructive pulmonary disease (COPD; ICD-9-CM 490–496), and lower body weight (ICD-9-CM 783.2) before the end date [18]. We also investigated drug effects and collected the history of oral steroid, bisphosphonates, hormone replacement therapy (HRT), vitamin D supplements, and aromatase inhibitors prescription before the end date. The oral steroid users was defined subjects had ever used the oral steroid at least one month [19]. Besides, the bisphosphonates, HRT, vitamin D supplements, and aromatase inhibitors were considered for adjustment, when subjects ever used for one dose before the study end date.

### Ethics statement

The NHIRD encrypts patient personal information to protect privacy and provides researchers with anonymous identification numbers associated with relevant claims information, including sex, date of birth, medical services received, and prescriptions. Therefore, patient consent is not required to access the NHIRD. This study was approved to fulfill the condition for exemption by the Institutional Review Board (IRB) of China Medical University (CMUH104-REC2-115). The IRB also specifically waived the consent requirement.

### Statistical analysis

We presented the mean and standard deviation (SD) for age and percentages for sex, age groups, occupation, comorbidities, and each type of drug used. We used the chi-squared test and Student’s t-test to assess the differences in the distribution between the RT and non-RT cohorts. The incidence–density of fragility fracture, osteoporosis / incident fragility fracture were calculated as the number of patients with incident fragility fracture, osteoporosis / fragility fracture divided by the sum of person–years (per 1000 person–years). We also measured the cumulative incidence curves of incident fragility fracture, osteoporosis / fragility fracture for both cohorts by using the Kaplan–Meier method. We used the log-rank test to assess the different incidence curves. We demonstrated the incidence–density between the RT and non-RT cohorts after stratifying the data on the basis of sex, age, occupation, comorbidities, oral steroid used, follow-up period (<2 and ≥2 years), and oral steroid use. The incidence rate ratio (IRR) of incident fragility fracture, osteoporosis / fragility fractures for these variables between the two cohorts was assessed using the Poisson regression model. The multivariable Cox proportional hazards model was used to estimate hazard ratios (HRs) and 95% and 98% confidence intervals (CIs) for the risk of incident fragility fracture, osteoporosis /fragility fracture between the two cohorts. The Bonferroni adjustment was used in multiple comparisons.

All the statistical analyses were performed using the SAS 9.4 statistical package (SAS Institute Inc., NC, USA). We used the R software (R Foundation for Statistical Computing, Vienna, Austria) to plot the cumulative incidence of both cohorts. A *p-value < 0*.*05* and (*p-value < 0*.*02*) was considered significant in the 2-tailed tests performed this study.

## Results

This study included 6612 patients with RT and 13220 normal controls from 1999 to 2005 (Table 1). The frequency-matched cohorts demonstrated a similar age and sex distribution (*p-value* > 0.05), in which 69.7% were men and 43.5% were ≧65 years old. Except for hypertension, celiac disease and Chron’s disease, the percentage of comorbidities in the RT cohort was greater than that in the non-RT cohort. RT cohort used the oral steroid (51.5%) or bisphosphonates (1.53%) was more common than comparison cohort.

**Table 1:** Comparison of demographics and comorbidity between with and without respiratory tuberculosis cohorts.

	Respiratory tuberculosis				
	No (N = 13220)		Yes (N = 6612)		
	n	%	n	%	*p*-value
**Sex**					0.99
Women	4008	30.3	2004	30.3	
Men	9212	69.7	4608	69.7	
**Age, year**					0.99
<35	2274	17.2	1137	17.2	
35–65	5196	39.3	2598	39.3	
≥65	5750	43.5	2877	43.5	
Mean (SD)^#^	56.7	20.2	56.9	20.3	0.64
**Occupation**					<0.001
White collar	5685	43.0	2468	37.3	
Blue collar	4839	36.6	2641	39.9	
Others	2696	20.4	1503	22.7	
**Comorbidity**					
Hyperlipidemia	3439	26.0	1576	23.8	<0.001
Hypertension	6446	48.8	3283	49.7	0.24
Diabetes	2909	22.0	1793	27.1	<0.001
Pneumonia	4712	35.6	4019	60.8	<0.001
Live cirrhosis	3565	27.0	2376	35.9	<0.001
IHD	3597	27.2	2038	30.8	<0.001
Stroke	3319	25.1	1806	27.3	<0.001
COPD	5793	43.8	4982	75.4	<0.001
ESRD	183	1.38	170	2.57	<0.001
Alcohol-related illness	374	2.83	447	6.76	<0.001
Hyperparathyroidism	12	0.09	18	0.27	0.002
Celiac disease	0	0.00	1	0.02	-
Chron’s disease	236	1.79	133	2.01	0.27
Lower body weight	323	2.44	286	4.33	<0.001
**Treatment**					
Oral steroid	4340	32.8	3402	51.5	<0.001
Bisphosphonates	76	0.57	101	1.53	<0.001
HRT	1218	9.21	651	9.85	0.15
Vitamin D supplements	21	0.16	12	0.18	0.71
Aromatase inhibitors	13	0.10	10	0.15	0.30

Chi-square test; ^#^ Student’s t-test; White collar: civil services, institution workers, enterprise, business and industrial administration personnel; Blue collar: farmers, fishermen, vendors, and industrial laborers; Others: retired, unemployed, and low-income populations; HRT, Hormone Replacement Therapy; IHD, ischemia heart disease; COPD, chronic obstructive pulmonary disease; ESRD, end-stage renal disease

**Fig 2:**
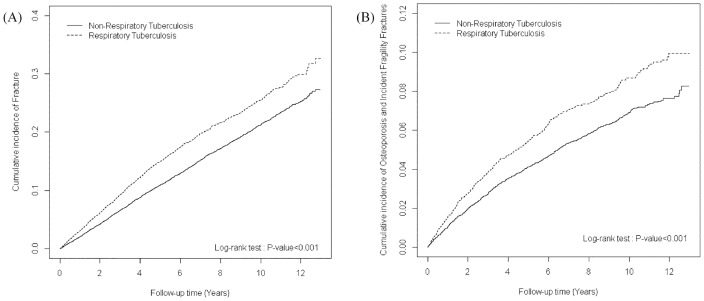
The cumulative incidence of incident bone fracture (A) and osteoporosis / fragility fracture (B) between non-respiratory tuberculosis cohort (solid line) and respiratory tuberculosis cohort (dashed line).

The cumulative incidence of incident fragility fracture (Fig 2A, log-rank *P < 0*.*001)* were significantly higher for patients in the RT cohort than for participants without RT. During the 44517 and 107232 person-years follow-up, the overall incidence density of fragility fractures was 1.69-fold significantly higher in RT patients than in the non-RT cohort (2.02 vs 1.11 per 1000 person-y), with an adjusted HR = 1.69 (95% CI = 1.26–2.28; 98% CI = 1.18–2.44) (Table 2). Stratified by sex, men with RT had a 57% increased fragility fracture risk compared to the non-RT cohort. The RT cohort was significantly associated with a higher risk of fragility fracture compared with the non-RT cohort for aged of 65–75 years (adjusted HR = 1.76, 95% CI = 1.12–2.75; 98% CI = 1.02–3.03) and ≧75 years (adjusted HR = 1.94, 95% CI = 1.18–3.19; 98% CI = 1.06–3.56). The adjusted HR of fragility fracture was higher in RT cohort regardless of white collar (adjusted HR = 2.19, 95% CI = 1.21–3.97; 98% CI = 1.06–4.52). The risk of fragility fracture was observed in patients with comorbidities (adjusted HR = 1.90, 95% CI = 1.43–2.52; 98% CI = 1.34–2.68) in the RT cohort than in the non-RT cohort. Stratified by oral steroid, RT patients with oral steroid or without oral steroid had an increased fragility fracture risk compared to the non-RT cohort. The adjusted HR of fragility fracture was significantly higher in the more than two follow-up year (adjusted HR = 1.67, 95% CI = 1.16–2.40; 98% CI = 1.07–2.59).

**Table 2:** Incidence and adjusted hazard ratio of Incident fragility fractures stratified by sex, age, comorbidity, oral steroid used and follow-up time between with and without respiratory tuberculosis cohorts.

	Respiratory tuberculosis								
		No			Yes			Compared to non-respiratory tuberculosis	
Variables	Event	PY	Rate	Event	PY	Rate	IRR (95% CI)	Adjusted HR^†^ (95% CI)	Adjusted HR^†^ (98% CI)
**Overall**	119	107232	1.11	90	44517	2.02	1.82(1.66, 2.00)***	1.69(1.26, 2.28)***	1.69(1.18, 2.44)**
**Sex**									
Women	46	33713	1.36	25	15002	1.67	1.22(1.02, 1.46)***	0.98(0.58, 1.65)	0.98(0.52, 1.85)
Men	73	73519	0.99	65	29515	2.20	2.22(1.98, 2.48)***	1.57(1.10, 2.23)**	1.57(1.02, 2.41)**
P for interaction									0.06
**Age, year**									
<65	21	67869	0.31	17	30751	0.55	1.79(1.56, 2.05)***	0.98(0.49, 1.98)	0.98(0.42, 2.30)
65–75	51	23387	2.18	42	8699	4.83	2.21(1.84, 2.66)***	1.76(1.12, 2.75)**	1.76(1.02, 3.03)**
≥75	47	15976	2.94	31	5066	6.12	2.08(1.72, 2.52)***	1.94(1.18, 3.19)**	1.94(1.06, 3.56)**
P for interaction									0.92
**Occupation**									
White collar	29	48453	0.60	24	18665	1.29	2.15(1.84, 2.50)***	2.19(1.21, 3.97)**	2.19(1.06, 4.52)**
Blue collar	59	38758	1.52	37	16878	2.19	1.44(1.23, 1.68)***	1.37(0.88, 2.13)	1.37(0.80, 2.35)
Others	31	20021	1.55	29	8974	3.23	2.09(1.72, 2.54)***	1.76(1.01, 3.06)*	1.76(0.90, 3.45)
P for interaction									0.99
**Comorbidity**									
No	4	24565	0.16	1	3705	0.27	1.66(1.19, 2.30)***	2.97(0.31, 28.4)	2.97(0.19, 46.4)
Yes	115	82667	1.39	89	40812	2.18	1.57(1.42, 1.74)***	1.90(1.43, 2.52)***	1.90(1.34, 2.68)**
P for interaction									0.60
**Oral steroid**									
No	64	73083	0.88	34	23253	1.46	1.22(1.09, 1.37)***	1.82(1.16, 2.87)**	1.82(1.05, 3.17)**
Yes	55	34149	1.61	56	21264	2.63	1.38(1.22, 1.55)***	1.63(1.10, 2.42)**	1.63(1.01, 2.64)**
P for interaction									0.52
**Follow-up, year**									
<2	35	25420	1.38	31	11455	2.71	1.97(1.77, 2.18)***	1.78(1.05, 2.99)*	1.78(0.94, 3.36)
≥2	84	81813	1.03	59	33062	1.78	1.74(1.57, 1.92)***	1.67(1.16, 2.40)**	1.67(1.07, 2.59)**

PY: person-year; Rate: incidence rate (per 1,000 person-years); IRR: incidence rate ratio; Adjusted HR^†^: multiple analysis including age, sex, occupation, drug of oral steroid, bisphosphonates, hormone replacement therapy (HRT), vitamin D supplements, and aromatase inhibitors and each comorbidity [including hyperlipidemia, hypertension, diabetes, pneumonia, live cirrhosis, ischemia heart disease (IHD), stroke, chronic obstructive pulmonary disease (COPD), end-stage renal disease (ESRD), alcohol-related illness, hyperparathyroidism, celiac disease, Chron’s disease, and lower body weight];

*p<0.05,

**p<0.02,

***p<0.001

The overall incidence of osteoporosis / fragility fracture was 9.61 per 1000 person–years and 7.29 per 1000 person–years in the RT and non-RT cohorts, respectively (Table 3). Fig 2B shows that the cumulative incidence of osteoporosis / fragility fracture was higher in the RT cohort than in the non-RT cohort (log-rank test < 0.001) at the end of the follow-up period. After adjustment for age, sex, drug use and comorbidities, the RT cohort was observed to be at a significantly higher risk of osteoporosis / fragility fracture compared with the non-RT cohort (adjusted HR = 1.42, 95% CI = 1.25–1.61; 98% CI = 1.21–1.65). The risk of osteoporosis was presented 1.36-fold (95% CI = 1.18–1.56; 98% CI = 1.14–1.61) in RT cohort. The RT cohort (except patients aged <65 years) was significantly associated with a higher risk of osteoporosis / fragility fracture compared with the non-RT cohort, particularly at the age of 65–75 years and ≧75 years. The adjusted HR of osteoporosis / fragility fracture was higher in RT cohort regardless of white collar (adjusted HR = 1.57, 95% CI = 1.24–1.99; 98% CI = 1.18–2.10), and others (adjusted HR = 1.72, 95% CI = 1.34–2.22; 98% CI = 1.27–2.34).

**Table 3:** Incidence and adjusted hazard ratio of osteoporosis / fragility fracture stratified by sex, age, comorbidity, oral steroid used and follow-up time between with and without respiratory tuberculosis cohorts.

	Respiratory tuberculosis								
		No			Yes		Compared to non-respiratory tuberculosis		
Variables	Event	PY	Rate	Event	PY	Rate	IRR (95% CI)	Adjusted HR^†^ (95% CI)	Adjusted HR^†^ (98% CI)
**Overall**	782	107232	7.29	428	44517	9.61	1.32(1.21, 1.43)***	1.42(1.25, 1.61)***	1.42(1.21, 1.65)**
Osteoporosis (ICD-9-CM: 7330)	663		6.18	338		7.59	1.23(1.12, 1.34)***	1.36(1.18, 1.56)***	1.36(1.14, 1.61)**
**Sex**									
Women	354	33713	10.5	189	15002	12.6	1.20(1.04, 1.39)*	1.11(0.92, 1.34)	1.11(0.89, 1.40)
Men	428	73519	5.82	239	29515	8.10	1.39(1.26, 1.54)***	1.15(0.97, 1.36)	1.15(0.94, 1.41)
P for interaction								0.34	
**Age, year**									
<65	210	67869	3.09	129	30751	4.19	1.36(1.21, 1.52)***	1.03(0.81, 1.30)	1.03(0.77, 1.37)
65–75	284	23387	12.1	169	8699	19.4	1.60(1.37, 1.87)***	1.50(1.22, 1.85)***	1.50(1.17, 1.93)**
≥75	288	15976	18.0	130	5066	25.7	1.42(1.21, 1.68)***	1.49(1.19, 1.86)***	1.49(1.14, 1.96)**
P for interaction								0.74	
**Occupation**									
White collar	236	48453	4.87	121	18665	6.48	1.33(1.16, 1.52)***	1.57(1.24, 1.99)***	1.57(1.18, 2.10)**
Blue collar	359	38758	9.26	184	16878	10.9	1.18(1.03, 1.34)*	1.16(0.95, 1.40)	1.16(0.92, 1.46)
Others	187	20021	9.34	123	8974	13.7	1.47(1.24, 1.74)***	1.72(1.34, 2.22)***	1.72(1.27, 2.34)**
P for interaction								0.48	
**Comorbidity**									
No	62	24565	2.52	5	3705	1.35	0.53(0.35, 0.82)**	1.11(0.44, 2.81)	1.11(0.36, 3.43)
Yes	720	82667	8.71	423	40812	10.4	1.19(1.09, 1.30)***	1.48(1.31, 1.68)***	1.48(1.28, 1.72)**
P for interaction								0.38	
**Oral steroid**									
No	516	73083	7.06	200	23253	8.60	1.22(1.09, 1.37)***	1.40(1.17, 1.67)***	1.40(1.13, 1.73)**
Yes	266	34149	7.79	228	21264	10.7	1.38(1.22, 1.55)***	1.46(1.21, 1.76)***	1.46(1.16, 1.83)**
P for interaction								0.73	
**Follow-up, year**									
<2	257	25419	10.1	162	11455	14.1	1.40(1.28, 1.53)***	1.59(1.28, 1.97)***	1.28(1.08, 1.52)**
≥2	525	81813	6.42	266	33062	8.05	1.25(1.15, 1.37)***	1.33(1.14, 1.57)***	1.67(1.07, 2.59)**

PY: person-year; Rate: incidence rate (per 1,000 person-years); IRR: incidence rate ratio; Adjusted HR^†^: multiple analysis including age, sex, occupation, drug of oral steroid, bisphosphonates, hormone replacement therapy (HRT), vitamin D supplements, and aromatase inhibitors and each comorbidity [including hyperlipidemia, hypertension, diabetes, pneumonia, live cirrhosis, ischemia heart disease (IHD), stroke, chronic obstructive pulmonary disease (COPD), end-stage renal disease (ESRD), alcohol-related illness, hyperparathyroidism, celiac disease, Chron’s disease, and lower body weight];

*p<0.05,

**p<0.02,

***p<0.001

The statistically significant increased risk of osteoporosis / fragility fracture was observed in patients with comorbidities (adjusted HR = 1.48, 95% CI = 1.31–1.68; 98% CI = 1.28–1.72) in the RT cohort. The RT cohort without oral steroid use was at a 1.40-fold increased risk of osteoporosis / fragility fracture compared with the non-RT cohort without oral steroid use (adjusted HR = 1.40, 95% CI = 1.17–1.67; 98% CI = 1.13–1.73), and the RT cohort was at a 1.46-fold increased osteoporosis / fragility fracture risk compared with the non-RT cohort with oral steroid use (adjusted HR = 1.46, 95% CI = 1.21–1.76; 98% CI = 1.16–1.83). The adjusted HR of osteoporosis / fragility fracture was significantly higher in the first two follow-up year (adjusted HR = 1.59, 95% CI = 1.28–1.97; 98% CI = 1.08–1.52) and in later on (adjusted HR = 1.33, 95% CI = 1.14–1.57; 98% CI = 1.07–2.59).

The incidence rate of fragility fracture was higher for those with comorbidities of hypertension, diabetes, pneumonia, live cirrhosis, and IHD in both cohorts (Table 4). The risk of fragility fracture was higher among patients without hypertension (adjusted HR = 2.81, 95% CI = 1.44–5.51) than patients with hypertension (adjusted HR = 1.52, 95% CI = 0.97–2.36) (P for interaction = 0.02).

**Table 4:** Incidence and adjusted hazard ratio of fragility fracture stratified by different comorbidity in respiratory tuberculosis and non-respiratory tuberculosis groups.

	Respiratory tuberculosis						Compared to without respiratory tuberculosis		
	No			Yes					
Variables	Event	PY	Rate	Event	PY	Rate	IRR (95% CI)	Adjusted HR^†^ (95% CI)	Adjusted HR^†^ (98% CI)
**Hyperlipidemia**									
No	90	78246	1.15	66	32997	2.00	1.77(1.59, 1.96)***	1.73(1.22, 2.46)**	1.73(1.13, 2.66)**
Yes	29	28986	1.00	24	11520	2.08	2.11(1.77, 2.52)***	2.17(1.22, 3.86)**	2.17(1.08, 4.37)**
P for interaction								0.52	
**Hypertension**									
No	31	57441	0.54	37	24315	1.52	2.86(2.50, 3.27)***	2.81(1.62, 4.89)***	2.81(1.44, 5.51)**
Yes	88	49792	1.77	53	20202	2.62	1.51(1.33, 1.71)***	1.52(1.05, 2.18)*	1.52(0.97, 2.36)
P for interaction								0.02	
**Diabetes**									
No	85	84771	1.00	66	33583	1.97	1.99(1.79, 2.20)***	1.86(1.30, 2.66)***	1.86(1.21, 2.87)**
Yes	34	22462	1.51	24	10934	2.19	1.49(1.25, 1.78)***	1.69(0.97, 2.95)	1.69(0.85, 3.33)
P for interaction								0.31	
**Pneumonia**									
No	52	70796	0.73	26	19286	1.35	1.87(1.64, 2.14)***	2.25(1.36, 3.72)**	2.25(1.22, 4.15)**
Yes	67	36436	1.84	64	25231	2.54	1.40(1.23, 1.59)***	1.62(1.13, 2.34)**	1.62(1.04, 2.53)**
P for interaction								0.22	
**Live cirrhosis**									
No	81	78405	1.03	59	27834	2.12	2.08(1.86, 2.32)***	1.89(1.31, 2.74)***	1.89(1.21, 2.96)**
Yes	38	28828	1.32	31	16683	1.86	1.44(1.23, 1.70)***	1.66(0.99, 2.78)	1.66(0.88, 3.11)
P for interaction								0.38	
**IHD**									
No	70	80293	0.87	51	32385	1.57	1.84(1.65, 2.05)***	1.98(1.32, 2.96)**	1.98(1.21, 3.24)**
Yes	49	26939	1.82	39	12132	3.21	1.79(1.53, 2.11)***	1.66(1.06, 2.59)*	1.66(0.96, 2.86)
P for interaction								0.71	
**ESRD**									
No	119	105958	1.12	89	43658	2.04	1.85(1.69, 2.02)***	1.80(1.33, 2.43)***	1.80(1.25, 2.59)**
Yes	0	1274	0	1	859	1.16	-	-	-
P for interaction								0.97	
**Alcohol-related illness**									
No	118	104094	1.13	83	41430	2	1.79(1.63, 1.96)***	1.72(1.27, 2.34)***	1.72(1.19, 2.50)**
Yes	1	3138	0.32	7	3086	2.27	7.67(4.11, 14.3)***	8.67(0.84, 80.1)	8.67(0.50, 149.9)
P for interaction								0.11	
**Hyperparathyroidism**									
No	118	107120	1.1	90	44375	2.03	1.87(1.71, 2.05)***	1.83(1.36, 2.47)***	1.83(1.27, 2.64)**
Yes	1	112	8.95	0	142	0	-	-	-
P for interaction								0.96	
**Celiac disease**									
No	119	107232	1.11	90	44505	2.02	1.85(1.69, 2.03)***	1.81(1.35, 2.45)***	1.81(1.26, 2.61)**
Yes	0	0	-	0	12	0	-	-	-
P for interaction								-	
**Chron’s disease**									
No	115	105230	1.09	88	43519	2.02	1.88(1.72, 2.06)***	1.88(1.39, 2.54)***	1.88(1.30, 2.71)**
Yes	4	2002	2.00	2	997	2.01	1.00(0.53, 1.87)	0.12(0.01, 3.18)	0.12(0.002, 6.50)
P for interaction								0.38	
**Lower body weight**									
No	115	104556	1.10	85	42491	2.00	1.85(1.69, 2.03)***	1.83(1.35, 2.48)***	1.83(1.26, 2.65)**
Yes	4	2676	1.49	5	2026	2.47	1.62(0.99, 2.66)	1.70(0.33, 8.70)	1.70(0.23, 12.4)
P for interaction								0.97	

PY: person-year; Rate: incidence rate (per 1,000 person-years); IRR: incidence rate ratio; Adjusted HR^†^: multiple analysis including age, sex, occupation, drug of oral steroid, bisphosphonates, hormone replacement therapy (HRT), vitamin D supplements, and aromatase inhibitors and each comorbidity [including hyperlipidemia, hypertension, diabetes, pneumonia, live cirrhosis, ischemia heart disease (IHD), stroke, chronic obstructive pulmonary disease (COPD), end-stage renal disease (ESRD), alcohol-related illness, hyperparathyroidism, celiac disease, Chron’s disease, and lower body weight];

**p<0.02,

***p<0.001

Table 5 shows the osteoporosis / fragility fracture risk between the RT and non-RT cohorts stratified by different comorbidities. Our results consistently showed that the RT cohort was significantly associated with an increased risk of osteoporosis / fragility fracture compared with the non-RT cohort in the presence of different comorbidities, such as hyperlipidemia, hypertension, diabetes, pneumonia, live cirrhosis, and IHD. The RT cohort without lower body weight have the higher risk of osteoporosis / fragility fracture (adjusted HR = 1.41, 95% CI = 1.24–1.60; 98% CI = 1.20–1.65), compared with the non-RT cohort also.

**Table 5:** Incidence and adjusted hazard ratio of osteoporosis / fragility fracture stratified by different comorbidity in respiratory tuberculosis and non-respiratory tuberculosis groups.

	Respiratory tuberculosis						Compared to without respiratory tuberculosis		
	No			Yes					
Variables	Event	PY	Rate	Event	PY	Rate	IRR (95% CI)	Adjusted HR^†^ (95% CI)	Adjusted HR^†^ (98% CI)
**Hyperlipidemia**									
No	533	78246	6.81	295	32997	8.94	1.31(1.19, 1.45)***	1.42(1.21, 1.66)***	1.42(1.17, 1.72)**
Yes	249	28986	8.59	133	11520	11.5	1.34(1.15, 1.57)***	1.43(1.14, 1.79)**	1.43(1.09, 1.88)**
P for interaction								0.72	
**Hypertension**									
No	259	57441	4.51	168	24315	6.91	1.53(1.36, 1.73)	1.50(1.21, 1.87)***	1.50(1.15, 1.96)****
Yes	523	49792	10.5	260	20202	12.9	1.23(1.10, 1.37)***	1.34(1.15, 1.58)***	1.34(1.11, 1.63)**
P for interaction								0.048	
**Diabetes**									
No	584	84771	6.89	297	33583	8.84	1.28(1.17, 1.41)***	1.39(1.20, 1.63)***	1.39(1.16, 1.68)**
Yes	198	22462	8.82	131	10934	12	1.36(1.16, 1.59)***	1.42(1.12, 1.80)**	1.42(1.07, 1.89)**
P for interaction								0.71	
**Pneumonia**									
No	452	70796	6.38	159	19286	8.24	1.29(1.14, 1.46)***	1.52(1.26, 1.84)***	1.52(1.20, 1.92)**
Yes	330	36436	9.06	269	25231	10.7	1.18(1.05, 1.32)**	1.33(1.12, 1.58)**	1.33(1.08, 1.64)**
P for interaction								0.19	
**Live cirrhosis**									
No	541	78405	6.9	261	27834	9.38	1.36(1.23, 1.50)***	1.43(1.22, 1.68)***	1.43(1.18, 1.74)**
Yes	241	28828	8.36	167	16683	10	1.20(1.04, 1.38)*	1.37(1.11, 1.69)**	1.37(1.06, 1.77)**
P for interaction								0.87	
**IHD**									
No	465	80293	5.79	251	32385	7.75	1.34(1.21, 1.48)***	1.51(1.27, 1.79)***	1.51(1.23, 1.86)**
Yes	317	26939	11.8	177	12132	14.6	1.24(1.08, 1.43)**	1.30(1.07, 1.58)**	1.30(1.02, 1.64)**
P for interaction								0.26	
**ESRD**									
No	777	105958	7.33	419	43658	9.6	1.31(1.20, 1.42)***	1.40(1.23, 1.60)***	1.40(1.20, 1.64)**
Yes	5	1274	3.92	9	859	10.5	2.67(1.46, 4.88)**	2.24(0.56, 8.98)	2.24(0.41, 12.2)
P for interaction								0.13	
**Alcohol-related illness**									
No	769	104094	7.39	410	41430	9.9	1.34(1.23, 1.46)***	1.40(1.23, 1.59)***	1.40(1.19, 1.64)**
Yes	13	3138	4.14	18	3086	5.83	1.41(0.96, 2.07)	2.61(1.14, 5.95)*	2.61(0.96, 7.12)
P for interaction								0.35	
**Hyperparathyroidism**									
No	780	107120	7.28	427	44375	9.62	1.32(1.22, 1.43)***	1.42(1.25, 1.61)***	1.42(1.21, 1.66)**
Yes	2	112	17.9	1	142	7.06	0.39(0.07, 2.35)	-	-
P for interaction								0.29	
**Celiac disease**									
No	782	107232	7.29	428	44505	9.62	1.32(1.22, 1.43)***	1.42(1.25, 1.61)***	1.42(1.21, 1.65)**
Yes	0	0	-	0	11.7	0	-	-	
P for interaction								-	
**Chron’s disease**									
No	766	105230	7.28	420	43519	9.65	1.33(1.22, 1.44)***	1.42(1.25, 1.62)***	1.42(1.22, 1.67)**
Yes	16	2002	7.99	8	997	8.02	1.00(0.56, 1.80)	0.85(0.31, 2.33)	0.85(0.25, 2.90)
P for interaction								0.41	
**Lower body weight**									
No	768	104556	7.35	413	42491	9.72	1.32(1.22, 1.44)***	1.41(1.24, 1.60)***	1.41(1.20, 1.65)**
Yes	14	2676	5.23	15	2026	7.4	1.42(0.91, 2.19)	2.03(0.86, 4.82)	2.03(0.71, 5.82)
P for interaction								0.74	

PY: person-year; Rate: incidence rate (per 1,000 person-years); IRR: incidence rate ratio; Adjusted HR^†^: multiple analysis including age, sex, occupation, drug of oral steroid, bisphosphonates, hormone replacement therapy (HRT), vitamin D supplements, and aromatase inhibitors and each comorbidity [including hyperlipidemia, hypertension, diabetes, pneumonia, live cirrhosis, ischemia heart disease (IHD), stroke, chronic obstructive pulmonary disease (COPD), end-stage renal disease (ESRD), alcohol-related illness, hyperparathyroidism, celiac disease, Chron’s disease, and lower body weight];

*p<0.05,

**p<0.02,

***p<0.001

The RT cohort without oral steroid use was at a 1.87-fold increased risk of fragility fractures (adjusted HR = 1.87, 95% CI = 1.20–2.90; 98% CI = 1.09–3.19) compared with the non-RT cohort (Table 6). The RT cohort using oral steroid demonstrated a statistically significant increased risk of fragility fractures in men (adjusted HR = 1.89, 95% CI = 1.19–3.00; 98% CI = 1.07–3.32). Similar results were observed for osteoporosis / fragility fracture.

**Table 6:** Adjusted hazard ratio of fragility fractures and osteoporosis / fragility fracture found the associated with respiratory tuberculosis and drugs.

	Event	PY	Rate	Adjusted HR^†^ (95% CI)	Adjusted HR^†^ (98% CI)
**Fragility fractures**					
**Overall**					
Without respiratory tuberculosis	119	107232	1.11	1.00	1.00
With respiratory tuberculosis					
Without oral steroid	34	23253	1.46	1.87(1.20, 2.90)**	1.87(1.09, 3.19)**
With oral steroid	56	21264	2.63	1.58(1.08, 2.31)*	1.58(0.99, 2.51)
**Men**					
Without respiratory tuberculosis	73	73519	0.99	1.00	1.00
With respiratory tuberculosis					
Without oral steroid	25	14837	1.68	2.26(1.32, 3.85)**	2.26(1.18, 4.33)**
With oral steroid	40	14678	2.73	1.89(1.19, 3.00)**	1.89(1.07, 3.32)**
**Women**					
Without respiratory tuberculosis	46	33713	1.36	1.00	1.00
With respiratory tuberculosis					
Without oral steroid	9	8416	1.07	1.25(0.56, 2.77)	1.25(0.47, 3.29)
With oral steroid	16	6586	2.43	1.09(0.55, 2.15)	1.09(0.47, 2.49)
Osteoporosis /fragility fractures					
**Overall**					
Without respiratory tuberculosis	782	107232	7.29	1.00	1.00
With respiratory tuberculosis					
Without oral steroid	200	23253	8.60	1.41(1.19, 1.67)***	1.41(1.14, 1.74)**
With oral steroid	228	21264	10.7	1.42(1.19, 1.70)***	1.42(1.14, 1.77)**
**Men**					
Without respiratory tuberculosis	428	73519	5.82	1.00	1.00
With respiratory tuberculosis					
Without oral steroid	107	14837	7.21	1.56(1.23, 1.98)***	1.56(1.17, 2.09)**
With oral steroid	132	14678	8.99	1.43(1.13, 1.81)**	1.43(1.08, 1.90)**
**Women**					
Without respiratory tuberculosis	354	33712	10.5	1.00	1.00
With respiratory tuberculosis					
Without oral steroid	93	8416	11.1	1.25(0.98, 1.61)	1.25(0.92, 1.70)
With oral steroid	96	6586	14.6	1.42(1.07, 1.89)*	1.42(1.00, 2.01)

PY, person-year; Rate, incidence rate (per 1,000 person-years); Adjusted HR^†^: multiple analysis including occupation, drug of oral steroid, bisphosphonates, hormone replacement therapy (HRT), vitamin D supplements, and aromatase inhibitors and each comorbidity [such as hyperlipidemia, hypertension, diabetes, pneumonia, live cirrhosis, ischemia heart disease (IHD), stroke, chronic obstructive pulmonary disease (COPD), end-stage renal disease (ESRD), alcohol-related illness, hyperparathyroidism, celiac disease, crohn disease, and lower body weight];

*p<0.05,

**p<0.02,

***p<0.001

## Discussion

A published meta-analysis including 167 studies evaluated the risk factors for a low bone marrow density (BMD)-related fracture in men and women, and reported age (>70 years), low body weight [body mass index (BMI) <20 to 25 kg/m^2^], weight loss (>10%), physical inactivity, prolonged corticosteroid use, and previous osteoporotic fracture as high risk factors [20]. An additional 102 studies assessed 15 other proposed risk factors but provided insufficient evidence in support of a male population [20]. This study suggested that the incidence of fragility fracture (adjusted HR = 1.69), osteoporosis / fragility fracture (adjusted HR = 1.42) in the RT cohort is higher than in the non-RT cohort, irrespective of age, sex, steroids use and comorbidities.

Respiratory tuberculosis is one of the diseases characterized granuloma formation which was controlled by cellular immune reactions, interferon-gamma (INF-gamma) which mediate inflammatory reactions increased in the tuberculosis [21]. Expression levels of IFN-gamma have a positive correlation to bone fracture [22]. The cytokine IFN-gamma stimulates neopterin release. High levels of neopterin were associated with increased hip fracture risk [23]. These previous finding imply that the RT associated with the bone fracture, even without comorbidities or steroid use. Holloway et al. study in 3 to 5% of active cases of tuberculosis [24] with bone fracture (e.g. compression fracture) in accordance with our study [25]. However, these hypothesis warrant further research.

Vitamin D deficiency and a low calcium intake were risk factors for osteoporosis observed in men. A study conducted by Ho-Pham et al suggested that vitamin D deficiency was also reported to be a risk factor for tuberculosis in men but not in women [26]. The dual effect of vitamin D deficiency on RT and osteoporosis may confound the association between RT and bone fracture. These factors support the higher risk of the fragility fracture (adjusted HR = 1.57, *P for interaction = 0*.*06*) observed in a male population than in female belonging to the RT cohort. Dehydroepiandrosterone (DHEA) and testosterone levels were profoundly reduced in the RT cohort along with a modest increase in cortisol and estradiol levels [27]. The low DHEA increased the RANAL activity owing to the decrease of the ratio of OPG / RANKL mRNA in osteoblasts [28], the high cortisol inhibit the OPG activity, thus resulting in the osteoporosis [8]. Wang NB et al, they found the osteoporosis with low BMD in the PTB patients also [29]. BMD changes were more marked in patients with RT than in those of the same age group with other chronic lung diseases agree this finding also [30]. The osteoporosis contribute to the bone fracture. Therefore, the adjusted HR of the RT cohort for fragility fracture was higher than that of the non-RT cohort, particularly in men (for men, adjusted HR = 1.57, *p <0*.*001*; for women, adjusted HR = 0.98; *p>0*.*05*).

Vitamin D deficiency was common in both underweight and normal-weight patients, but an association between vitamin D deficiency and reduced femur neck T scores was indicated only in the underweight patients with RT [31]. The median 25(OH)D level increased after tuberculosis treatment. In Taiwan, patients with RT receive antituberculosis (anti-TB) treatment [16] or anti-TB with a short-term low dose steroid may increase the BMI of patients with RT and malnutrition [32]. The BMD of Taiwanese women shows a positive relationship to body weight and BMI in the femoral neck fracture group [33]. Similarly, patients with inhaled steroid use [34] may have the less incidence of the osteoporosis [35]. Therefore, RT cohort with steroid use and anti-TB drug may have an increased BMI [35], 25(OH)D level [31], and BMD [35]; these may explain the RT cohort with the lower body weight reveal no significant risk of the incident fragility fracture (aHR = 1.70, *p value >0*.*05*) or osteoporosis / fragility fracture (aHR = 2.03, *p value >0*.*05*). These hypotheses warrant research.

The Nanjundaiah et al. report, Lewis rats by injection of heat-killed *M*. *tuberculosis* H37Ra (Mtb). The antigen-presenting cells (APCs) took up the microbial antigens. APCs process antigens and then present these Mtb to antigen-specific T cells. These antigen-primed T cells then migrate into the joints, and release proinflammatory cytokines (osteopontin, tumor necrosis factor-α, interleukin) locally leading to arthritic inflammation. These cytokines also stimulate the production of RANKL/MCSF, which activates osteoclasts producing cathepsin K (Cat K) and matrix metalloproteases (MMPs) resulting in bone damage such as osteoporosis [36]. Similarly, sinomenine could attenuate osteoclast formation and Mtb-induced bone loss by mediating RANKL signaling pathways in Li et al study support the RT presenting as systemic inflammation and contribute to the osteoporosis in our study [37].

Women who lived in institutions and had osteoporosis, RT, and cardiac diseases (hypertension) were at risk of a contralateral hip fracture after the initial hip fracture [38]. Activity restriction led to an increased bone resorption in hospitalized women, which possibly affected the risk of osteopenia and osteoporosis [39]. In our study, the patients with the hypertension (aHR = 1.34, *p value <0*.*05*) and IHD (aHR = 1.30, *p value <0*.*05*) were association with the osteoporosis / fragility fracture in RT cohort. Meanwhile, women in the RT cohort with the oral steroid use are at an increased risk of osteoporosis / fragility fracture (aHR = 1.42, 95% CI; *p value <0*.*05*) than the non-RT cohort. These finding in line with previous study. Yet, this result need the random control trial for confirmation.

Previous studies have reported that metabolic-syndrome-related diseases such as hyperlipidemia, hypertension, diabetes, ischemic heart diseases, ESRD [10], and liver cirrhosis [11] were more common in patients with osteoporotic fracture. This study also demonstrated that metabolic diseases increased the risk of bone fracture, osteoporosis / fragility fracture. Meanwhile, alcohol-related illness were associated with the bone fractures, osteoporosis / fragility fracture in RT cohort also. Previous studies have rarely reported pneumonia as a risk factor of osteoporosis. In this study, we observed that both acute pneumonia (viral and bacterial) and chronic respiratory infectious diseases (RT) increased the risk of fragility fracture (aHR = 1.69), osteoporosis / fragility fracture (aHR = 1.42).

Respiratory infections were frequently observed in elderly patients with malnutrition. The combined effect of malnutrition, respiratory infection, and physical inactivity contributes to osteoporosis / incident fragility fracture [40]. In our study, we found that a higher incidence of bone fracture, osteoporosis / fragility fracture among the frequently hospitalized patients with RT even without low body weight, or without steroid use; compared with patients without RT. This study increases physicians awareness of incident bone fracture associated with RT.

## Limitations

Several limitations must be considered when interpreting these findings. The NHIRD does not provide detailed lifestyle information such as smoking, BMI, and physical activity, which were potential confounding factors in this study. However, anti-TB treatment and lifestyle modification of patients with RT may implicate these factors in accelerated bone fracture or osteoporosis / fragility fracture observed in RT. Additionally, information on osteoporosis severity scales such as disease activity, functional impairment, and physical damage may lead to underdiagnosis of this metabolic syndrome. Insufficient drug data such as oral antidiabetic drugs to adjust for the outcomes of interest could be another limitation. The evidence derived from this retrospective cohort study is generally of lower methodological evidence than that from randomized controlled trials because a retrospective cohort study is subject to many biases due to lack of the necessary adjustments for possible confounding factors (such as lack of the important data like serum levels OPG, RANKL, osteoclast activity markers and bone formation markers; and individual data of the histological or densitometric changes in bone). The Nanjundaiah and Li et al. study were experimental study in animals. Therefore, further investigation with a prospective and randomized controlled study design to reveal the cause-effect between RT and bone fracture or osteoporosis / fragility fracture would be worthwhile. In addition, lack of BMD data to do the optimal diagnosis of osteoporosis in the NHIRD is the study limitation. However, NHIRD covers a highly representative sample of Taiwan’s general population because the reimbursement policy is universal and operated by a single-buyer, the government in Taiwan. All insurance claims should be scrutinized by medical reimbursement specialists and peer review according to the standard diagnosed criteria in the study. If these doctors or hospitals make wrong diagnoses or coding, they will be punished with a lot of penalties. Therefore, the diagnoses of osteoporosis based on ICD-9 codes in this study were highly reliable. In addition, fracture reported here is based on ICD codes, not X-ray report. We can’t distinguish between atraumatic and traumatic fractures. Meanwhile, Underlying causes of pathological fractures include osteoporosis, metastatic bone tumor, osteomyelitis, Paget’s disease, disuse atrophy, hyperparathyroidism, and nutritional or congenital disorders [Coding for Fractures: For The Record Vol. 20 No. 24 P. 28 (Great Valley Publishing Co., Inc)]. After we did analyses, we didn’t find the coding of the [ICD-9-CM -198.5, Secondary malignant neoplasm of bone and bone marrow; ICD-9-CM—170.9, Malignant neoplasm of bone and articular cartilage, site unspecified; ICD-9-CM-731.0, Osteitis deformans without mention of bone tumor] in the RT cohort. The impact of these confounding factors on the fragility fractures needs further investigation. Finally, when performing several tests, increasing the family-wise error rate (FWER, the probability of rejecting at least one null hypothesis erroneously). In statistics, FWER is the probability of making one or more false discoveries, or typeΙerrors when performing multiple hypotheses tests. The Bonferroni adjustment will always provide strong control of the family-wise error rate [41]. If the number of comparisons in the test becomes large, the test may become too conservative and no longer allows us to find anything significant [42].

## Strength

The nationwide population-based longitudinal cohort study used to investigate the risk factors of incident bone fracture, osteoporosis / fragility fracture in an Asian population with RT was the strength of this study. We investigated drug effects such as oral steroid of the impact on the osteoporosis. We have replaced the smoking history data with the comorbidity which were associated with smoking such as IHD, stroke and COPD for adjustment. Meanwhile the life style such as drinking (alcohol-related disease) and patients received anti-osteoporosis drugs were included for analysis also. Furthermore, the study included participants from the general population representative of a true population, thereby improving the generalizability of our results. Even the BMD unavailable in this study; the policy of diagnosis of the osteoporosis is well established in Taiwan [43]. The coding the osteoporosis under the “guideline for the prevention and treatment of osteoporosis in Taiwan” at most healthcare system [44]. This multidisciplinary system may avoid the diagnosis bias.

## Conclusion

The Respiratory tuberculosis associated with the incident fragility fracture, either in men or absence of oral steroid use

## Supporting Information

S1 STROBE Checklist (DOC)

## Abbreviations

aHR: adjusted hazard ratio
APCs: antigen-presenting cells
Cat K: cathepsin K
CI: confidence interval
HCV: hepatitis C virus
HRT: hormone replacement therapy
INF-gamma: interferon-gamma
LHID 2000: Longitudinal Health Insurance Database 2000
MMPs: matrix metalloproteases
Mtb: *M*. *tuberculosis*
NHIRD: National Health Insurance Research Database
NHI: National Health Insurance
NHRI: National Health Research Institutes
OPG: osteoprotegerin
PTB: pulmonary tuberculosis
PTH: Parathyroid hormone
RANKL: receptor activator of nuclear factor kappa-B ligand
RT: respiratory tuberculosis

## References

[1] Burge R, Dawson-Hughes B, Solomon DH, Wong JB, King A, Tosteson A. Incidence and economic burden of osteoporosis-related fractures in the United States, 2005–2025. J Bone Miner Res. 2007;22: 465–475. 10.1359/jbmr.061113 17144789

[2] Shriraam V, Mahadevan S, Anitharani M, Selvavinayagam, Sathiyasekaran B. National health programs in the field of endocrinology and metabolism—Miles to go. Indian J Endocrinol Metab. 2014;18: 7–12. 10.4103/2230-8210.126521 24701424 PMC3968736

[3] Sale JE, Beaton D, Bogoch E. Secondary prevention after an osteoporosis-related fracture: an overview. Clin Geriatr Med. 2014;30: 317–332. 10.1016/j.cger.2014.01.009 24721371

[4] Osteoporosis. Information from your family doctor. Am Fam Physician. 2009;79: 201–202. 19202967

[5] Hodgson SF, Watts NB, Bilezikian JP, Clarke BL, Gray TK, Harris DW, et al American Association of Clinical Endocrinologists medical guidelines for clinical practice for the prevention and treatment of postmenopausal osteoporosis: 2001 edition, with selected updates for 2003. Endocr Pract. 2003;9: 544–564. 10.4158/EP.9.6.544 14715483

[6] Trouvin AP, Goeb V. Receptor activator of nuclear factor-kappaB ligand and osteoprotegerin: maintaining the balance to prevent bone loss. Clin Interv Aging. 2010;5: 345–354. 10.2147/CIA.S10153 21228900 PMC3010170

[7] Sambrook P, Cooper C. Osteoporosis. Lancet. 2006;367: 2010–2018. 10.1016/S0140-6736(06)68891-0 16782492

[8] Romas E. Clinical applications of RANK-ligand inhibition. Intern Med J. 2009;39: 110–116. 10.1111/j.1445-5994.2008.01732.x 19356186

[9] McGowan B, Bennett K, Marry J, Walsh JB, Casey MC. Patient profile in a bone health and osteoporosis prevention service in Ireland. Ir J Med Sci. 2012;181: 511–515. 10.1007/s11845-012-0806-9 22373588

[10] Lai SW, Liao KF, Lai HC, Tsai PY, Lin CL, Chen PC, et al Risk of major osteoporotic fracture after cardiovascular disease: a population-based cohort study in Taiwan. J Epidemiol. 2013;23: 109–114. 10.2188/jea.JE20120071 23269126 PMC3700249

[11] Dong HV, Cortes YI, Shiau S, Yin MT. Osteoporosis and fractures in HIV/hepatitis C virus coinfection: a systematic review and meta-analysis. AIDS. 2014;28:2119–31. 10.1097/QAD.0000000000000363 24977441 PMC4940983

[12] Pattamapaspong N, Muttarak M, Sivasomboon C. Tuberculosis arthritis and tenosynovitis. Semin Musculoskelet Radiol. 2011;15: 459–469. 10.1055/s-0031-1293492 22081281

[13] Spiegel DA, Singh GK, Banskota AK. Tuberculosis of the Musculoskeletal System. Techniques in Orthopaedics. 2005;20: 167–178.

[14] Yen YF, Yen MY, Lin YP, Shih HC, Li LH, Chou P, et al Directly observed therapy reduces tuberculosis-specific mortality: a population-based follow-up study in Taipei, Taiwan. PLoS ONE. 2013;8: e79644 10.1371/journal.pone.0079644 24278152 PMC3838349

[15] Pietri M, Lucarini S. The orthopaedic treatment of fragility fractures. Clin Cases Miner Bone Metab. 2007;4: 108–116. 22461210 PMC2781236

[16] Curtis JR, Taylor AJ, Matthews RS, Ray MN, Becker DJ, Gary LC, et al "Pathologic" fractures: should these be included in epidemiologic studies of osteoporotic fractures? Osteoporos Int. 2009;20: 1969–1972. 10.1007/s00198-009-0840-2 19184268 PMC2766025

[17] Yang Y, Du F, Ye W, Chen Y, Li J, Zhang J, et al Inpatient cost of treating osteoporotic fractures in mainland China: a descriptive analysis. Clinicoecon Outcomes Res. 2015;7: 205–212. 10.2147/CEOR.S77175 25926747 PMC4403816

[18] Yeh CC, Wang HH, Chou YC, Hu CJ, Chou WH, Chen TL, et al High risk of gastrointestinal hemorrhage in patients with epilepsy: a nationwide cohort study. Mayo Clin Proc. 2013;88: 1091–1098. 10.1016/j.mayocp.2013.06.024 24012412

[19] Evans DJ. The use of adjunctive corticosteroids in the treatment of pericardial, pleural and meningeal tuberculosis: do they improve outcome? Respir Med. 2008;102: 793–800. 10.1016/j.rmed.2008.01.018 18407484

[20] Liu H, Paige NM, Goldzweig CL, Wong E, Zhou A, Suttorp MJ, et al Screening for osteoporosis in men: a systematic review for an American College of Physicians guideline. Ann Intern Med. 2008;148: 685–701. 18458282 10.7326/0003-4819-148-9-200805060-00009

[21] Kim M, Ahn JH, Moon HS, Park SH, Song JS. The Changes of Serum Level of Tumor Necrosis Factor-Alpha, Gamma-Interferon and Soluble-Intercellular Adhesion Molecule-1 Relating to the Progression and Treatment of Patients with Pulmonary Tuberculosis. Tuberc Respir Dis. 1998;45: 1167–1177.

[22] Chen H, Cheng C, Li M, Gao S, Li S, Sun H. Expression of TNF-alpha, IFN-gamma, TGF-beta, and IL-4 in the spinal tuberculous focus and its impact on the disease. Cell Biochem Biophys. 2014;70: 1759–1764. 10.1007/s12013-014-0125-z 25326857

[23] Apalset EM, Gjesdal CG, Ueland PM, Øyen J, Meyer K, Midttun Ø, et al Interferon gamma (IFN-gamma)-mediated inflammation and the kynurenine pathway in relation to risk of hip fractures: the Hordaland Health Study. Osteoporos Int. 2014;25: 2067–2075. 10.1007/s00198-014-2720-7 24817202 PMC4099528

[24] Holloway KL, Henneberg RJ, de Barros Lopes M, Henneberg M. Evolution of human tuberculosis: a systematic review and meta-analysis of paleopathological evidence. Homo. 2011;62: 402–458. 10.1016/j.jchb.2011.10.001 22093291

[25] Holloway KL, Link K, Ruhli F, Henneberg M. Skeletal lesions in human tuberculosis may sometimes heal: an aid to palaeopathological diagnoses. PLoS ONE. 2013;8: e62798 10.1371/journal.pone.0062798 23638146 PMC3634763

[26] Ho-Pham LT, Nguyen ND, Nguyen TT, Nguyen DH, Bui PK, Nguyen VN, et al Association between vitamin D insufficiency and tuberculosis in a Vietnamese population. BMC Infect Dis. 2010;10: 306 10.1186/1471-2334-10-306 20973965 PMC2978214

[27] Rey AD, Mahuad CV, Bozza VV, Bogue C, Farroni MA, Bay ML, et al Endocrine and cytokine responses in humans with pulmonary tuberculosis. Brain Behav Immun. 2007;l21: 171–179. 10.1016/j.bbi.2006.06.005 16890403

[28] Wang YD, Wang L, Li DJ, Wang WJ. Dehydroepiandrosterone inhibited the bone resorption through the upregulation of OPG/RANKL. Cell Mol Immunol. 2006;3: 41–45. 16549048

[29] Wang NB, Wang HX, Gao JX. Therapeutic effect of exercise therapy on bone mineral density and low back pain in pulmonary tuberculosis patients with osteoporosis. Chinese Journal of Clinical Rehabilitation. 2005;9: 176–177.

[30] Savula MM, Kravchenko NS, Slivka IuI. [Bone mineral density in some lung diseases]. Probl Tuberk Bolezn Legk. 2004;45–47. 15379043

[31] Førli L, Halse J, Haug E, Bjørtuft Ø, Vatn M, Kofstad J, et al Vitamin D deficiency, bone mineral density and weight in patients with advanced pulmonary disease. J Intern Med. 2004;256: 56–62. 10.1111/j.1365-2796.2004.01337.x 15189366

[32] Dooley DP, Carpenter JL, Rademacher S. Adjunctive corticosteroid therapy for tuberculosis: a critical reappraisal of the literature. Clin Infect Dis. 1997;25: 872–887. 9356803 10.1086/515543

[33] Yang TS, Chen YR, Chen YJ, Chang CY, Ng HT. Osteoporosis: prevalence in Taiwanese women. Osteoporos Int. 2004;15: 345–347. 10.1007/s00198-003-1509-x 14872301

[34] Liu SF, Kuo HC, Liu GH, Ho SC, Chang HC, Huang HT, et al Inhaled corticosteroids can reduce osteoporosis in female patients with COPD. Int J Chron Obstruct Pulmon Dis. 2016;11: 1607–1614. 10.2147/COPD.S106054 27478374 PMC4951067

[35] Yanik B, Ayrim A, Ozol D, Koktener A, Gokmen D. Influence of obesity on bone mineral density in postmenopausal asthma patients undergoing treatment with inhaled corticosteroids. Clinics (Sao Paulo). 2009;64: 313–318. 19488588 10.1590/S1807-59322009000400008 PMC2694454

[36] Nanjundaiah SM, Astry B, Moudgil KD. Mediators of inflammation-induced bone damage in arthritis and their control by herbal products. Evid Based Complement Alternat Med. 2013;2013: 518094 10.1155/2013/518094 23476694 PMC3582100

[37] Li X, He L, Hu Y, Duan H, Li X, Tan S, et al Sinomenine suppresses osteoclast formation and Mycobacterium tuberculosis H37Ra-induced bone loss by modulating RANKL signaling pathways. PLoS ONE. 2013;8: e74274 10.1371/journal.pone.0074274 24066131 PMC3774760

[38] Liu S, Zhu Y, Chen W, Sun T, Cheng J, Zhang Y. Risk factors for the second contralateral hip fracture in elderly patients: a systematic review and meta-analysis. Clin Rehabil. 2015;29:285–94. 10.1177/0269215514542358 25027445

[39] Vanderspank D, Bernier SM, Sopper MM, Watson P, Mottola MF. Activity restriction increases deoxypyridinoline excretion in hospitalized high-risk pregnant women. Biol Res Nurs. 2014;16: 7–15. 10.1177/1099800412463120 23079370

[40] Kanis JA, McCloskey EV, Johansson H, Cooper C, Rizzoli R, Reginster JY, et al European guidance for the diagnosis and management of osteoporosis in postmenopausal women. Osteoporos Int. 2013;24: 23–57. 10.1007/s00198-012-2074-y 23079689 PMC3587294

[41] Armstrong RA. When to use the Bonferroni correction. Ophthalmic Physiol Opt. 2014;34: 502–508. 10.1111/opo.12131 24697967

[42] Perneger TV. What’s wrong with Bonferroni adjustments. BMJ. 1998;316: 1236–1238. 9553006 10.1136/bmj.316.7139.1236 PMC1112991

[43] Hwang JS, Chan DC, Chen JF, Cheng TT, Wu CH, Soong YK, et al Clinical practice guidelines for the prevention and treatment of osteoporosis in Taiwan: summary. J Bone Miner Metab. 2014;32: 10–16. 10.1007/s00774-013-0495-0 24068612

[44] Yeh MC, Weng SF, Shen YC, Chou CW, Yang CY, Wang JJ, et al Increased Risk of Sudden Sensorineural Hearing Loss in Patients With Osteoporosis: A Population-based, Propensity Score-matched, Longitudinal Follow-Up Study. J Clin Endocrinol Metab. 2015;100: 2413–2419. 10.1210/jc.2014-4316 25879512

